# Supplementary material for: Opioids modulate an emergent rhythmogenic process to depress breathing
Source: eLife. 2019 Dec 16;8:e50613. doi: 10.7554/eLife.50613 (PMC6938398; doi:10.7554/eLife.50613)
Supplement: Supplementary file 1. [file elife-50613-supp1.pdf]

Supplementary file 1. Correlation of burst and burstlet parameters and preBötC *f*.

Comparison	Condition	r^2	p-value	n
burstlet fraction vs. preBötC *f* (Related to Figure 1A-C)	9/1.5	0.0026	0.5	6
	10 nM DAMGO	0.26	0.1	6
	30 nM DAMGO	0.031	0.4	6
	Change in correlation		0.8	6
burstlet fraction vs. preBötC *f* (Related to Figure 1D-F)	30 nM DAMGO+ 100 µM picrotoxin/1 µM strychnine	0.00002	0.5	13
preBötC burst amp. vs. preBötC *f* (Related to Figure 1D-F)	30 nM DAMGO+ 100 µM picrotoxin/1 µM strychnine	0.1	0.1	13
burstlet fraction vs. preBötC *f* (Related to Figure 1G-I)	3/1	0.21	0.1	7
	10 nM DAMGO	0.38	0.1	7
	30 nM DAMGO	0.0022	0.5	7
	Change in correlation		0.6	7
burstlet fraction vs. preBötC *f* (Related to Figure 2A, B)	3/1*	0.71	0.005	8
	Cd^{2+}	0.059	0.3	8
	Cd^{2+}+DAMGO	0.037	0.3	8
	Change in correlation		0.9	8
burstlet fraction vs. preBötC *f* (Related to Figure 4C, D)	3/1	0.073	0.3	6
	500 nM SP	0.53	0.05	6
	Change in correlation		0.3	6
burstlet fraction vs. preBötC *f* (Related to Figure 4E, F)	3/1	0.11	0.1	13
	100 µM picrotoxin/1 µM strychnine	0.04	0.3	13
	Change in correlation		0.1	13

*, $p<0.05$
